# Some Pathological Features of Lungs from Domestic and Wild Ruminants with Single and Mixed Protostrongylid Infections

**DOI:** 10.4061/2010/741062

**Published:** 2010-04-11

**Authors:** Mariana Stancheva Panayotova-Pencheva, Marin Tsvyatkov Alexandrov

**Affiliations:** Institute of Experimental Pathology and Parasitology, Bulgarian Academy of Sciences, Acad. G. Bonchev Street, Block 25, 1113 Sofia, Bulgaria

## Abstract

Lungs of 40 ruminants from Bulgaria with natural small lungworm (Nematoda: Protostrongylidae) infections were investigated, including 16 goats, 15 sheep, 7 mouflons, and 2 chamois. *Muellerius capillaris, M. tenuispiculatus, Cystocaulus ocreatus, Neostrongylus linearis,* and *Protostrongylus brevispiculum* infections were predominantly associated with nodular lesions, and *Protostrongylus rufescens, Protostrongylus hobmaieri* and *Protostrongylus rupicaprae* were associated with extensive lesions located mainly along the length of the large bronchi. The extent of lung abnormalities was most severe in the sheep. Alveolitis, parasite granulomas, damage of the alveolar septa, hyperplasia of the lung associated lymphoid tissue, and sclerosis of the parenchyma were found upon microscope examinations. In the goats compared to the sheep and mouflons, the terminal bronchi, bronchioles, and alveoli were more affected than the interstitium. Our research shows that the pathological lesions in the lungs of ruminants infected with protostrongylids depend on both the helminth and the host species. To our knowledge, this work is the first to provide data on the pathomorphological lesions in mouflon lungs infected with protostrongylids.

## 1. Introduction

Protostrongylidoses are widespread helminthoses in domestic and wild ruminants causing a serious health problem for a great number of countries all over the world. Their etiological agents are the so-called small lungworms (Nematoda: Protostrongylidae). These infections are mainly associated with respiratory disturbances [1, 2], development of focal pneumonia [3, 4], and secondary bacterial infections of the lungs [5–7]. Loss of weight, lower animal productivity, reduced numbers of offspring, abortions, or neonatal deaths, and increased mortality [3, 8–11] have also been linked with many protostrongylidoses. The pathologic features of these ailments have been studied in detail, but the data obtained show that various kinds of inflammatory reactions can be found within the lesions. Therefore, based on the fact that the types of lung damage produced by protostrongylids are numerous and vary in severity and extent in the different studies [8, 12–19] the aim of the present study was to analyze the most frequent pathological lesions in the lungs of goats, sheep, mouflons, and chamois from Bulgaria attributed to different protostrongylid species in order to achieve a more detailed characterization of the particular parasitic infections in the different hosts.

## 2. Materials and Methods

Lungs of forty ruminants from different regions of Bulgaria, including 16 goats (*Capra aegagrus f. domestica* L.), 15 sheep (*Ovis ammon f. domestica* L.), 7 mouflons (*Ovis musimon* L.) and 2 chamois (*Rupicapra rupicapra* L.), were investigated. The lungs of the domestic ruminants were collected during standard postmortem examination at several slaughter houses from Veliko Tarnovo and Sofia provinces. Information about the animals' age was obtained by the owners of the flocks. The lungs of wild ruminants were obtained after selective hunting of animals inhabiting wildlife-breeding reserves in Dobrich and Smolyan provinces. All procedures were approved by the Institute of Experimental Pathology and Parasitology Ethics Committee and complied with the requirements of Ordinance No 22/14.12.2005 on reducing to a minimum suffering of animals at the time of slaughter or killing (Promulgated in SG 22 of 14 Dec 2005 and transposing Directive 93/119/EC on the protection of animals at the time of slaughter or killing) and European Union guidelines for the accommodation and care of animals used for experimental and other scientific purposes (2007/526/EC). The materials were transported immediately to the laboratory with the exception of 20 lungs (8 of goats, 5 of sheep, 5 of mouflons, and 2 of chamois) which were deep frozen and transported later.

Firstly, a topographic sketch of the superficial abnormalities in the lungs was drawn. After that routine necropsies of the lungs were performed. The helminths within the particular lesion were found and their species were determined under the microscope. Aiming to find the small, hardly visible worms located in the bronchioles and alveoli, the abnormal lung tissues were examined as follows: 1-2 cm^3^ parts from the lung lesions were selected and boiled in 40% lactic acid in a water bath for 1.5 hour. After that small (2-3 mm) pieces of those parts were compressed and observed under a light microscope. In this way the lungworms became brighter and sexual structures of the male specimens were clearly visible. The identification of the helminths observed was carried out on the basis of their morphometric characteristics [5].

The locality, consistency, colour, formation of nodules, presence of abscesses, presence of other nonparasitic lesions, and dissemination were taken into consideration during the evaluation of the macroscopic lesions in the infected lungs. According to their dissemination, macroscopic lesions were subjectively classified as follows: slight (small, hardly visible lesions), mild (affecting up to 1/6 of the lung surfaces), severe (affect from 1/6 to 1/2 of the lung surfaces), and very severe (affecting over 1/2 of the lung surfaces).

 Small organ pieces (0.5 × 2 × 2 cm) were obtained from the helminthologicaly analyzed (but not boiled) tissue lesions and processed for histopathological examination. They were fixed in 10% phosphate buffered formalin, embedded in paraffin, cut into sections of 5 to 10 *μ*m thick, and stained with haematoxylin and eosin according to standard technique.

## 3. Results

The parasitic lesions established in the present study were located within the caudal lung lobes and were disseminated mainly within the dorsal subpleural parenchyma. The results of the macroscopic examinations as well as the species of the hosts and protostrongylids established in each animal are presented in Tables 1, 2 and 3.

**Figure 1:**
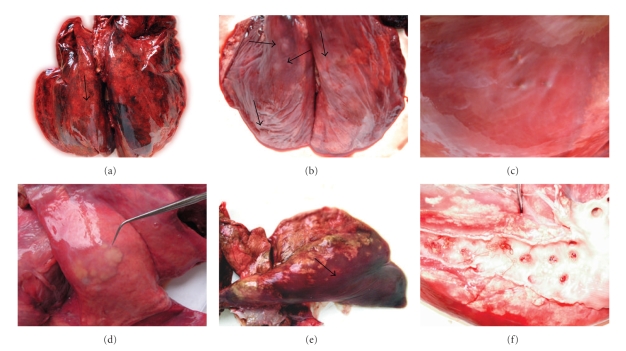
Lungs of ruminants infected with protostrongylids. (a) Mouflon, lungs, *M. capillaris* infection, dark-grey to black, congested areas, (b) Mouflon, lungs, *M. capillaris* infection. Well-formed, firm, grey nodules, (c) Mouflon, lung, *M. capillaris* infection. Small, hard nodules (1–5 mm) under the lung pleura, (d) Sheep, lung, *C. ocreatus* infection, Slightly prominent, well differentiated from the surrounding tissue area, (e) Goat, right lung, *P. rufescens* infection, dark-red to grey, congested area, (f) Goat, lung, *P. rufescens* infection, adult worms in bronchi.

The lung lesions in goats infected with *M. capillaris* were nodular, firm, and gray. In the younger animals small, grayish areas on the dorsal surface of the caudal lung lobes were found. Rarely larger congested, gray areas, situated close to the lung pleura, were observed in the goats. The lesions in the lungs of sheep infected with *M. capillaris* were more severe, regardless of the animals' age. In the most cases they were not formed as nodules and were firm, grey to black areas affecting a large part of the lung surfaces. Diffuse congestion of the lung tissue was found when those abnormalities were cross-sectioned. The lesions in the lungs of mouflons infected with *M. capillaris* were of several types—grey patches on the lung surfaces of different size, extensive, diffuse, dark-grey to black congested areas (Figure 1(a)), and well-formed, firm, grey nodules (Figure 1(b)). Small hard nodules of a diameter of 1–5 mm under the lung pleura also were observed in the mouflon lungs (Figure 1(c)). Nodular lesions were observed in the lungs of chamois and sheep infected with *M. tenuispiculatus*, *N. linearis,* and *P. brevispiculum*. Adult *C. ocreatus* were found in nodules having a different structure (Figure 1(d)). They were well differentiated from the surrounding tissue, slightly prominent above the serous surface of the visceral pleura, but not very dense and of normal or soft consistency. They contained internal cavities filled with a caseous substance. The macroscopic abnormalities in the lungs of ruminants infected with larger species of the genus *Protostrongylus* consisted primarily of extensive congestion of the tissue along the largest bronchi and were dark-red to grey (Figure 1(e)). In these cases adult helminths were found in the dissected bronchi (Figure 1(f)).

The results of the histological investigations, concerning abnormalities in the bronchi, alveoli, and alveolar septa as well as the progress of specific changes in the lung parenchyma and interstitium around the adult worms, eggs, and larvae of the parasites are shown in Table 4.

**Figure 2:**
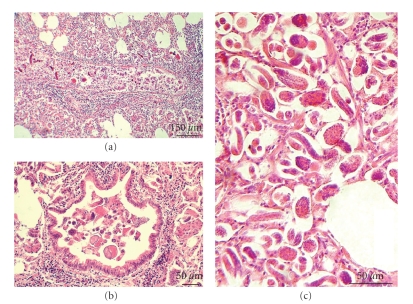
Goats, lungs infected with protostrongylids. (a) A nodule with *M. capillaris*, bronchial lumen with larvae and a desquamation of the bronchial epithelium. (b) A lobular lesion with *M. capillaris*, *P. rufescens,* and *P. hobmaieri*, bronchial lumen with adult parasites, larvae, alveolar macrophages, and a slight peribronchial lymphoid hyperplasia. (c) A nodule with *M. capillaris*, alveoli with parasite eggs and first stage larvae without thickening of the alveolar septa.

The abnormalities in the lungs of goats varied in severity in the affected lobules and acini. The bronchi and bronchioles contained desquamated epithelial cells, alveolar macrophages, neutrophils, and parasite forms in different stages, sometimes encircled by slight lymphoid hyperplasia (Figures 2(a) and 2(b)). Many alveoli also contained a great number of parasite forms (eggs and first stage larvae) and inflammatory exudate consisting of alveolar macrophages and neutrophils. However, in many of these cases a thickening of the alveolar septa was not observed (Figure 2(c)).

**Figure 3:**
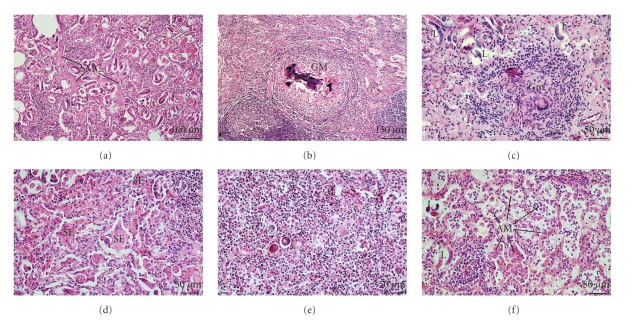
(a) Sheep, lung, a nodule with *M. capillaris,* alveoli with parasite forms, smooth muscle cell (SMC) hyperplasia and a thickening of the alveolar septa. (b) Sheep, lung, a nodule with *M. capillaris*, parasite granuloma (Gm) with calcification in the necrotic centre and lymphoid hyperplasia in the periphery. (c) Mouflon, lung, a nodule with *M. capillaris*. Granuloma with giant cells (Gm). First stage larva (L). (d) Sheep, lung, a lobular lesion with *P. hobmaieri*. Serous alveolitis (SE). (e) Sheep, lung, a lobular lesion with *P. hobmaieri*. Alveoli with epitheloid and giant cells. (f) Mouflon, lung, a nodule with *M. capillaris*. Alveolar macrophages (AM) in the alveolar lumen (macrophage alveolitis).

**Figure 4:**
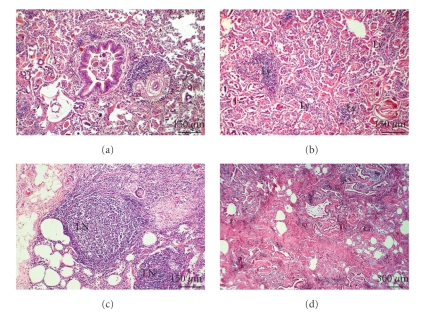
Sheep, lungs, infected with protostrongylids. (a) A nodule with *M. capillaris*. Peribronchial lymphoid hyperplasia. (b) A lobular lesion with *P. hobmaieri*. Disseminated lymphoid hyperplasia (Ly). (c) A lobular lesion with *P. hobmaieri*. Intralobular lymphoid hyperplasia, formation of lymphoid nodules (LNs). (d) A lobular lesion with *M. capillaris*. Sclerosis of the parenchyma with a lymphocyte infiltration of the sclerotic areas.

 Similar abnormalities were found in the lungs of sheep and mouflons. Additionally, the thickening of the alveolar septa as a consequence of hyperplasia of the connective tissue and smooth muscle cells was regularly observed (Figure 3(a)). Parasite granulomas consisting of a necrotic centre, calcification, peripheral lymphoid hyperplasia, and giant cells were observed frequently (Figures 3(b) and 3(c)). Development of serous, giant cell, and macrophage alveolitis was demonstrated (Figures 3(d), 3(e), and 3(f)). In sheep and mouflons, marked hyperplasia of the lymphoid tissue was also observed. Hyperplastic lymphoid structures were situated both close around the bronchi and in the lung interstitium and sometimes were spread to whole lobules (Figures 4(a), 4(b), and 4(c)). In some animals, sclerosis of the parenchyma with a lymphocyte infiltration of the sclerotic areas was observed (Figure 4(d)).

## 4. Discussion

Many pathological features of the lungs in domestic and wild ruminants with protostrongylid infections observed in the present study have been described by other researchers [8, 13, 14, 16, 17, 19–24]. However, limited attention has been paid to the parasite-host interaction within the inflammatory foci. The inflammatory response and the variety of lymphoid hyperplasia observed in the present study clearly indicate that interesting protostrongylid-host interactions are taking place in the protostrongylid infected lungs.

Comparing our results with the data from other studies in goats with protostrongylid infections we find both similarities and differences. Our observations confirm Svarc's [25] conclusions that the application of infective *M. capillaris* larvae causes a nodular form of muelleriosis in goats but it can be associated also with an exudative reaction. To a certain extent our results coincide with those of Nimmo [24] who found dark coloured areas mostly on the dorsal surfaces of the caudal lung lobes in 2-year-old animals with a *Muellerius *sp. infection. Such lesions we observed in kids under 6 months old. In older goats we observed the formation of nodules, which is missing according to Nimmo [24]. The author [24] found only congestion of the lung tissue and asserts that diffuse interstitial pneumonia is the most obvious histological change in goats with *Muellerius *sp. infection, although according to other studies that finding is typical mostly of sheep. Nimmo [24] associates the diffuse interstitial pneumonia with thickening of the alveolar septa, which is due to fibromuscular hyperplasia and mononuclear cell infiltration. However, according to our data many of the alveolar septa remain without visible morphological abnormalities despite the presence of a large number of parasites in the alveolar lumen. According to the results of Valero et al. [13] the lesions in goats associated with *M. capillaris* infection are nodular, disseminated mostly on the caudo-dorsal aspects of the lungs and their prevalence increase with age. The data of Berrag et al. [8] are similar. Histologically Berrag et al. [8] describe a variable degree of inflammatory cell reaction. In that respect their data are in accordance with ours. Regarding the macroscopic lesions our results were similar to those of Berrag and Cabaret [17] who ascertain nodules in the case of *M. capillaris *infection and lobular lesions in *P. rufescens* infection. With respect to the dissemination of the lung abnormalities in animals of a particular age category, however, our results were different. Berrag and Cabaret [17] indicate that the surface of the lungs covered with protostrongylid lesions is larger in kids than in older goats but we found the opposite. That difference most likely is due to the increased sensitivity of individual nonimmune young animals investigated by the authors [17] affected for the first time in their life with a massive protostrongylid infection. 

Our studies show that the lesions in the sheep with *M. capillaris *infection are rarely nodular and represent extensive, diffusely congested, grey areas. There were some differences in the macroscopic lung lesions in goats and sheep. The lesions in sheep grew predominately into deep lung tissue whereas those in the goats were more superficial. Formation of firm, distinct, prominent nodules in sheep were observed mainly due to *N. linearis* and *P. brevispiculum* infections. As a whole the lungs of sheep were affected to a greater degree than those of the goats. The cases of slight and mild lung abnormalities in the sheep were few even in young animals. Severe abnormalities predominated and mixed infections were often connected with very severe abnormalities. Our results show that the microscopic abnormalities in the sheep lungs with *M. capillaris *infection are similar to those in goats. Furthermore, in sheep, thickening of the alveolar septa and formation of parasite granulomas varying in cell structure were observed. In the mixed infections, giant cell alveolitis and marked hyperplasia of the lymphoid tissue were observed. Beresford-Jones [20] describes irregularly disseminated, prominent, yellow-grey, diffuse lesions under the lung pleura penetrating into the parenchyma as well. These data support our results that the lung lesions in sheep with *M. capillaris* infection are mostly of a diffuse rather than nodular character. There is a similarity in the histological picture too. A thickening of the alveolar septa in the lungs of sheep with *M. capillaris* infection is observed both in the studies of Beresford-Jones [20] and in ours. Thomas et al. [21] found that 98% of lung lesions in sheep with* M. capillaris* infection affect the caudal lobes. However, they describe the established lesions as nodular and do not mention the more widespread ones which we found in many of the cases. Our results were in accordance with Svarc [22] who points out the presence of both types of lesions as well. According to Bouljihad et al. [16] the lesions in the lungs infected with *M. capillaris *and* P. rufescens* appear as red to brownish thickened areas. The lesions so described are similar to our findings only with regard to infection with *P. rufescens*, lesions observed also by Mansfield and Gamble [14] in sheep with a *P. rufescens* infection. Nodular lesions and the presence of capsules around them attributed to *C. ocreatus* infection also have been established by scanning electron microscopy [19].

The lesions in the lungs of mouflons with *M. capillaris* infection were both diffuse and nodular and in this case it was difficult to determine which predominated. Lesions associated with *N. linearis, C. ocreatus, *and* P. rufescens* infections were analogous to those in sheep. The lesions associated with *M. capillaris* in our work were similar to those established by Demartini and Davies [23] in other wild sheep species (*Ovis canadensis *L.) from Colorado in the United States. More typical findings in the mouflons' lungs were the small, clearly differentiated, very hard, grayish nodules under the lung pleura. Many hard, crystal-like structures and helminth parts were observed in these nodules under compression after boiling in lactic acid. We are unaware of data in the literature about the pathological lesions in mouflons with protostrongylid infections. The nodules mentioned above, however, were rather similar to the granulomas described by Beresford-Jones [20] in sheep infected with *M. capillaris *while McFadyean 1894 (citation after Beresford-Jones [20]) describes them as pseudotubercules. According to the criteria subjectively adopted by us the lung abnormalities in mouflons were categorized as mild. Severe abnormalities were observed only in one case of a mixed infection with 4 protostrongylid species. Very severe abnormalities were not found. The microscopic abnormalities in the mouflons were similar to those in sheep. The abnormalities in the bronchi were more frequent in the presence of *P. rufescens* infection. Often, in mixed infections, neutrophilic-macrophagic alveolitis and hyperemia of the septa were found. The granulomas formed had large areas of calcification and macroscopically corresponded to the small, hard nodules under the lung pleura.

The macroscopic lung lesions in the chamois were diffuse in the mixed *P. rupicaprae* and *N. linearis* infection and nodular in the *M. tenuispiculatus* and *N. linearis* infection. Small hard nodules such as those described in mouflons were found in chamois too. As a whole the lesions established by us were similar to those described in chamois by Svarc [26, 27] and Iacob et al. [28].

According to Stockdale, 1976, and Chitwood and Lichtenfels, 1972 (citation after Valero at al. [13]) different species of protostrongylids produce similar gross and microscopic lesions. However, our studies show some unique aspects of the pathomorphological lung lesions in ruminants depending on the nematode and host species. These lesions were analyzed in view of refining and correlating the pathomorphological and parasitological criteria required for the diagnosis of those infections and can be summarized as follows.

- In goats, the gross lung lesions associated with *M. capillaris* infections were mainly nodular, firm, and gray.
- Nodular lesions were also observed in the cases of *M. tenuispiculatus, C. ocreatus, N. Linearis, *and* P. brevispiculum *infections in the different ruminants*. P. rufescens, P. hobmaieri,* and *P. rupicaprae* produced large, diffuse dark-red macroscopic lesions, commonly situated along the large bronchi.
- In the lungs of goats infected with protostrongylids the alveoli, terminal bronchi and bronchioles were affected to a greater degree than the interstitium. The prevalence of parasitic lesions increased with age.
- Compared with goats, the lungs of sheep infected with protostrongylids were more severely affected, regardless of age. Severe abnormalities predominated. The microscopic abnormalities were considerable and variable.
- The lesions in the mouflon lungs were both diffuse and nodular. In contrast to goats, the lung interstitium of sheep and mouflons was more strongly affected.

In summary, our research shows that the pathological lesions in the lungs of ruminants infected with protostrongylids depend both on the helminth and the host species.

## Figures and Tables

**Table 1:** Macroscopic lesions and classification of the lung change severity in goats with natural protostrongylid infections.

Host age	Lung parasite	Lesions											Classification of macroscopic lesions
		Localization			Colour			Consistency		Nodules	Abscesses	OL	
		DACLL	VACLL	MACLL	G	DG-B	DR-G	N	F				
6 months	*Muellerius*	**+**			+			+					slight
	*capillaris*												
6 months	*M. capillaris*	**+**			+			+					slight
6 months	*M. capillaris*	+			+			+					slight
6 months	*M. capillaris*	+			+			+					slight
6 months	*M. capillaris*	+			+			+					slight
7 years	*M. capillaris*			+		+			+	+	+		mild
8 months	*M. capillaris*	+	+			+			+	+	+		severe
9 months	*M. capillaris*	+	+			+			+	+			mild
18 months	*M. capillaris*	+			+				+		+		severe
8 years	*M. capillaris*	+	+		+	+			+			+	very severe
6 years	*M. capillaris*	+	+		+				+	+		+	severe
3 years	*M. capillaris*	+	+		+	+			+	+	+		severe
	*Protostrongylus*												
	* hobmaieri*												
3 years	*M. capillaris*	+		+	+	+			+	+			severe
	*P. hobmaieri*												
2 years	*M. capillaris*	+					+		+	+			severe
	*P. rufescens*												
1 year	*M. capillaris*	+					+		+	+		+	very severe
	*P. rufescens*												
30 months	*M. capillaris*	+			+				+	+			very severe
	*P. hobmaieri*												
	*P. rufescens*												

DACLL: dorsal areas of the caudal lung lobes; VACLL: ventral areas of the caudal lung lobes; MACLL: mediastinal areas of the caudal lung lobes; G: grey; DG-B: dark-grey to black; DR-G: dark-red to grey; N: normal; F: firm; OL-other lesions (emphysema, cysts); + present sign; blank-absent sign.

**Table 2:** Macroscopic lesions and classification of the lung change severity in sheep with natural protostrongylid infections.

Host age	Lung parasite	Lesions											Classification of macroscopic lesions
		Localization			Colour			Consistency		Nodules	Abscesses	OL	
		DACLL	VACLL	MACLL	G	DG-B	DR-G	N	F				
<1 year	*M. capillaris*	**+**		**+**	+	+			+	+			mild
<1 year	*M. capillaris*	**+**			+	+			+				severe
<1 year	*M. capillaris*	+			+				+				Severe
<1 year	*M. capillaris*	+	+		+				+			+	mild
<1 year	*M. capillaris*	+			+			+					slight
8 years	*M. capillaris*	+			+				+		+	+	severe
ni	*M. capillaris*	+	+		+				+		+	+	severe
5 years	*M. capillaris*	+		+	+				+	+	+		severe
<1 year	*M. capillaris*	+	+	+			+		+	+			mild
6 years	*M. capillaris*	+	+	+	+				+	+	+		severe
	*P. brevispiculum*												
<1 year	*M. capillaris*	+			+					+		+	severe
	*P. hobmaieri*												
ni	*M. capillaris*	+	+		+		+		+	+	+		very severe
	*P. rufescens*												
ni	*M. capillaris*	+	+	+	+	+	+		+			+	very severe
	*P. rufescens*												
ni	*M. capillaris*	+			+			+	+	+	+		severe
	*C. ocreatus*												
	*N. linearis*												
<1 year	*M. capillaris*	+	+	+	+	+	+	+		+			very severe
	*C. ocreatus*												
	*N. linearis*												
	*P. rufescens*												

DACLL: dorsal areas of the caudal lung lobes; VACLL: ventral areas of the caudal lung lobes; MACLL: mediastinal areas of the caudal lung lobes; G: grey; DG-B: dark-grey to black; DR-G: dark-red to grey; N: normal; F: firm; OL: other lesions (emphysema, cysts); ni: there was not information; + present sign; blank-absent sign.

**Table 3:** Macroscopic lesions and classification of the lung change severity in mouflons and chamois with natural protostrongylid infections.

Host species	Lung parasite	Lesions								Classification of macroscopic lesions
		Localization		Colour		Consis-Tency		Nodules	Abscesses	
		DACLL	VACLL	G	DG–B	N	F			
mouflon	*M. capillaris*	**+**	**+**	+			+	+		Mild
mouflon	*M. capillaris*	**+**		+			+	+	+	Slight
mouflon	*M. capillaris*	+		+			+	+		Mild
mouflon	*M. capillaris*	+			+	+	+			Mild
mouflon	*M. capillaris*	+	+	+			+	+		Mild
	*N. linearis*									
mouflon	*M. capillaris*	+		+			+	+		Mild
	*N. linearis*									
mouflon	*M. capillaris*	+	+	+			+	+		Severe
	*N. linearis*									
	*C. ocreatus*									
	*P. rufescens*									
chamois	*P. rupicaprae*	+	+	+		+				Mild
	*N. linearis*									
chamois	*M. tenuispiculatus*	+		+			+	+		Mild
	*N. linearis*									

DACLL: dorsal areas of the caudal lung lobes; VACLL: ventral areas of the caudal lung lobes; G: grey; DG-B: dark-grey to black; N: normal; F: firm; + present sign; blank-absent sign.

**Table 4:** Microscopic abnormalities in the lungs of domestic and wild ruminants with natural protostrongylid infections.

			Host species					
		Goat	Goat	Sheep	Sheep	Sheep	Mouflon	Mouflon
	Number of animals	3	1	8	1	1	1	1

Location	Protostrongylid species	*M. capillaris*	*M. capillaris*	*M. capillaris*	*M. capillaris*	*M. capillaris*	*M. capillaris*	*M. capillaris*
			*P. rufescens*		*P. hobmaieri*	*C. ocreatus*		*C. ocreatus*
			*P. hobmaieri*			*N. linearis*		*N. linearis*
						*P. rufescens *		*P. rufescens*

In the bronchi	Presence of parasite forms	+	+	+	+	+	−	+
	Alveolar macrophages and neutrophils in the lumen	+	+	+	+	+	−	+
	Desquamation of the bronchial and bronchiolar epithelium	+	+	+	+	+	−	+

In the alveoli	Parasite forms	+	+	+	+	−	+	+
	Macrophage alveolitis	+	+	+	+	+	−	+
	Neutrophilic alveolitis	+	+	+	+	+	−	+
	Mixed macrophage and neutrophilic alveolitis	+	+	+	+	+	−	+
	Serous alveolitis	−	−	−	+	+	−	−
	Giant cell alveolitis	−	−	−	+	−	−	+
	Alveolar emphysema	−	−	+	+	−	−	+

In the alveolar septa	Thickening of the septa	−	−	+	+	+	+	−
	Hyperemia of the septa	−	−	−	−	−	+	−

In the interstitium	Giant cell reaction, infiltration with monocytes and eosinophils	−	−	−	−	−	−	−

Parasite granulomas	With disintegrated adult worm or larvae in the center	−	−	+	−	−	−	+
	With necrotic center in different stages of calcification	−	−	−	−	+	+	−
	With lymphoid hyperplasia	−	−	+	−	−	−	+
	With giant and epitheloid cells	−	−	−	+	−	+	+

Lymphoid hyperplasia	Peribronchial	+	+	+	+	+	−	+
	Perilobular	−	−	−	−	−	−	−
	Intralobular	−	−	−	+	+	−	−
	Disseminated	−	−	+	*+*	+	−	+

Sclerosis of the parenchyma	Diffuse sclerosis, infiltration with lymphocytes	−	−	+	+	−	+	−
